# Human Benefits of Animal Interventions for Zoonosis Control 

**DOI:** 10.3201/eid1304.060381

**Published:** 2007-04

**Authors:** Jakob Zinsstag, Esther Schelling, Felix Roth, Bassirou Bonfoh, Don de Savigny, Marcel Tanner

**Affiliations:** * Swiss Tropical Institute, Basel, Switzerland; † International Livestock Research Centre, Nairobi, Kenya; ‡ Institut du Sahel, Bamako, Mali

**Keywords:** human health, society, benefits, interventions, livestock, brucellosis, rabies, avian influenza, synopsis

## Abstract

Although industrialized countries have been able to contain recent outbreaks of zoonotic diseases, many resource-limited and transitioning countries have not been able to react adequately. The key for controlling zoonoses such as rabies, echinococcosis, and brucellosis is to focus on the animal reservoir. In this respect, ministries of health question whether the public health sector really benefits from interventions for livestock. Cross-sectoral assessments of interventions such as mass vaccination for brucellosis in Mongolia or vaccination of dogs for rabies in Chad consider human and animal health sectors from a societal economic perspective. Combining the total societal benefits, the intervention in the animal sector saves money and provides the economic argument, which opens new approaches for the control of zoonoses in resource-limited countries through contributions from multiple sectors.

## Abstract 2

Animal interventions to control zoonoses save money, even in resource-limited countries.

The economic aspects of controlling zoonoses are rapidly gaining attention in light of challenges, both well-known and new. Wildlife reservoirs of classical and emerging zoonoses (e.g., bovine tuberculosis) persist in many countries and substantially slow control efforts for livestock (*1*). The fast-growing demand for milk and meat in urban centers in resource-limited countries is leading to the intensification of livestock production systems, especially in periurban areas of these countries. However, because efficient zoonosis surveillance and food safety are lacking, the risk for zoonosis transmission is increasing, particularly in rapidly growing urban centers of resource-limited countries (*2*,*3*). Many countries in postcommunist transition face a sharp increase in zoonotic diseases resulting from the breakdown of government-run disease surveillance and control and weak private health and veterinary services (*4*).

Industrialized countries have responded rapidly to recent zoonosis outbreaks and contained them well (*5*), but many resource-limited and transitioning countries have not been able to respond adequately because they lack human and financial resources and have not sufficiently adapted public health surveillance. In industrialized countries, an important part of successful zoonosis control has been compensating farmers for culled livestock. However, many resource-limited countries would not be able to conduct such programs.

Most zoonoses are maintained in the animal reservoir but can cross over to humans as a result of different risk factors and behavioral traits. For example, brucellosis is transmitted to humans from direct contact with livestock or ingestion of unpasteurized milk or milk products; however, brucellosis is not transmitted from humans to livestock. Hence, elimination of zoonoses such as rabies, echinococcosis, and brucellosis is possible only by interventions that vigorously target animal reservoirs. Control of most zoonoses usually requires interventions outside the public health sector. When one considers health from a point of view independent of species, including humans, domestic animals, and wildlife, zoonoses are part of a broader ecologic concept of health systems (*6*–*8*). To attempt control, and possibly elimination, of zoonoses, benefits to public health and society need to be demonstrated, particularly in countries with scarce resources. We present examples from our work on brucellosis and rabies and demonstrate the circumstances for which zoonosis control would save money for resource-limited countries and likely reduce the occurrence of zoonoses worldwide. Avian influenza is discussed as an additional example.

## Diseases

### Brucellosis

In Mongolia and central Asian countries after democratic reform and the shift from dependence on the former Soviet Union in 1990, human brucellosis reemerged as a major, but preventable, disease (*9*). After consultations with experts, the World Health Organization (WHO) raised the question whether mass vaccinations of animals saved money for the public health sector. We used an animal-to-human transmission model to estimate the economic benefit, cost-effectiveness, and distribution of benefit (to society and the public health and agricultural sectors) of mass brucellosis vaccination of cattle and small ruminants (*10*). The intervention consisted of a planned 10-year annual livestock mass vaccination campaign using *Brucella melitensis* Rev-1 for small ruminants and *Brucella abortus* S19 for cattle. In a scenario of achieving 52% reduction of brucellosis transmission between animals, 51,856 human brucellosis cases could be averted, which would add up to a gain of 49,027 human disability-adjusted life years (DALYs; see Appendix). The human death rate from brucellosis is considered to be <2% (*11*) and was not assessed in this study (*4*). Estimated intervention costs were US $8.3 million, and the overall benefit was US $26.6 million (Table 1). This results in a present net value of $18.3 million and a benefit-to-cost ratio for society of 3.2 (95% confidence interval [CI] 2.27–4.37). If the costs of the intervention were shared between the sectors in proportion to the benefit to each, the public health sector would contribute 11%, with a cost-effectiveness ratio of $18 per case averted. However this ratio does not account for illness imposed by brucellosis. When the number of DALYs averted is assumed to be 49,027, the cost-effectiveness ratio is $19.1 per DALY averted (95% CI 5.3–486.8). If costs of vaccinating livestock are allocated proportionally to all benefits, the intervention is cost-saving and cost-effective for the agricultural and the public health sectors (*4*). With such an allocation of costs to benefits per sector, brucellosis control becomes one of the most cost-effective interventions (<$25 per DALY gained) in the public health sector, comparable to cost-effectiveness of vaccinating women and children or treating tuberculosis.

**Table 1:** Distribution of benefits and suggested allocation of intervention costs for livestock brucellosis mass vaccination campaign in Mongolia*†

	Cost	Benefit	Net present value‡	Benefit: cost ratio§
Total agriculture sector	5,174.9	16,611.6	11,436.7	3.2
Human health				
Public health sector				
Central government	1,009.4	3,240.3	2,230.9	3.2
Health Insurance Fund	0.0	0.0	0.0	
Patient				
Out-of-pocket contribution to health costs	1,669.3	5,358.7	3,689.4	3.2
Change in household income	1,103.7	3,542.8	2,439.1	3.2
Total overall human health	3,782.4	12,141.8	8,359.4	3.2
Total private sector	7,947.9	25,513.1	17,565.2	3.2
Total society	8,957.3	28,753.4	19,796.1	3.2

*Scenario for proportion of protected animals at 52% and discount rate at 5%. Costs are in Mongolian tugriks; 1 US$ ≈1,080 MNT in October 2000.  †Table reproduced from (*4*). ‡Benefits minus costs. §Benefits divided by costs (minimum 2.27, maximum 4.37).

### Rabies

Most human deaths from rabies occur in tropical resource-limited countries (*12*). In Africa and Asia, an estimated 24,000–70,000 persons die of rabies each year (*13*). The domestic dog is the main source of exposure and vector for human rabies (*14*). Rabies in humans can be prevented by appropriate postexposure prophylaxis, which is not, however, always available and affordable in resource-limited countries. Human rabies can also be prevented through vaccination of the animal vector. Again, particularly for countries with limited resources, we must ask whether it is cost-saving to the public health sector to prevent human rabies by vaccinating dogs. Bögel and Meslin showed that over 15 years in areas where the virus still circulates in the dog population, dog vaccination combined with postexposure treatment of dog-bite patients is more cost-effective than postexposure prophylaxis alone (*15*). However, in many countries, little is known about the real cost of mass vaccination of dogs, and quantitative data are urgently needed to evaluate the cost-effectiveness of different rabies control strategies in resource-limited countries; rabies control strategies in developing countries are currently under review by WHO (F. Meslin, pers. comm.). 

We performed a cost analysis of a pilot dog rabies vaccination campaign in which 3,000 dogs in N’Djaména, Chad, were vaccinated (*16*). The average cost per dog was US $2.14 to the public sector (for vaccine and logistics) and $0.97 to the private-sector dog owner, which brings the full cost to society to $3.11. If all 23,600 dogs in N'Djamena were vaccinated, the average cost per dog would fall to $1.48 for the public sector and $2.45 for society (Table 2). Private sector costs account for 31% of the cost to vaccinate 3,000 dogs and 40% of the cost to vaccinate 23,600 dogs (*17*). The above costs per vaccinated dog tally with the estimates by Bögel and Meslin (*15*). The cost estimates provided account for only 1 year of vaccination. In the year before this campaign (*16*,*17*), 69 persons were reported to have been exposed to 29 rabid dogs. If one assumes that human exposure could be avoided by mass vaccination of dogs, the cost-effectiveness of mass dog rabies vaccination would be $837 per averted human exposure ($57,774 per 69 averted exposures). If one assumes that about 16% of the exposed persons would actually become ill with rabies and die (*18*), the cost-effectiveness would be $57,774 per 11 averted deaths ($5,252 per averted death). But if we consider that reported human rabies cases in Africa underestimate the true number of human rabies cases by a factor of 10 to 100 (*18*), then the cost-effectiveness would be $52–$525 per averted death. Hence, mass vaccination of dogs is a comparatively inexpensive and ethical way to control the disease in animals and to prevent human exposure and illness, especially in resource-limited countries. More research is needed to assess the dynamics of dog-to-human rabies transmission and the frequency of revaccination programs needed because of turnover in dog populations and continued risk for reintroduction of rabies from outside sources to unvaccinated dogs.

**Table 2:** Cost per vaccinated dog extrapolated for the dog population of N’Djaména, Chad*†

Item	Total cost (US$)
Public sector	
Marginal	
Vaccine	14,368
Syringes and certificates	5,079
Fixed	
Furniture and small equipment‡	507
Staff§	8,425
Transportation¶	4,653
Information#	1,806
Total public sector	34,838
Private sector	
Lost work time	22,879
Total private sector	22,879
Total campaign	57,717

*Average population 23,600 dogs. †Table adapted with permission from (*17*). ‡Tables, chairs, coolers, ice, screens, muzzles, first aid materials. §Per diem for training, information campaign, vaccination program, lunches. ¶Car, small truck, fuel. #Campaign, poster distribution.

### Avian Influenza

The spread of highly pathogenic avian influenza is a global threat to all countries that have a poultry industry, semicommercial poultry production, or backyard poultry operations and has already caused enormous economic losses (*19*). Moreover, the risk for human pandemic influenza originating from highly pathogenic avian influenza in conjunction with human influenza A virus is very high, with an estimate of >100,000 deaths for the United States alone (*20*). To implement disease prevention and control measures, early identification of emerging patterns of disease is necessary and uses economic methods to determine which mix of measures is most cost-effective. Resource-limited countries in Africa are almost devoid of surveillance capacity and efficient early warning systems, which would be crucial. Surveillance of cross-border diseases cannot be restricted to countries that have the funds. High-income countries would ultimately benefit by providing funding for surveillance and control to low-income countries. Comprehensive economic assessment of this issue are, however, lacking so far.

## Awareness, Knowledge, and Information

Many countries, especially those with resource constraints and those in sub-Saharan Africa, lack information on the distribution of zoonotic diseases. Risks for zoonoses are considered negligible compared with those for diseases of higher consequence because the societal consequences of zoonoses are not recognized by the individual sectors. For example, outbreaks of Rift Valley fever in persons in Mauritania were mistakenly identified as yellow fever. The correct diagnosis was made only after public health services contacted livestock services, which informed them of abortions in cattle (*21*). In resource-limited and transitioning countries, many zoonoses are not controlled effectively because adequate policies and funding are lacking. However, transmission of zoonoses to humans can already be greatly reduced by health information and behavior. Authorities in Kyrgyz, for example, have started an information campaign to reduce brucellosis transmission to small-ruminant herders by encouraging them to wear gloves for lambing and to boil milk before consuming. Interventions in livestock should always be accompanied by mass information, education, and communication programs.

## Financing

Substantial evidence documents that the combined effects of human disease caused by zoonoses, as part of the neglected infectious diseases, are in the same range as the classical diseases of poverty such as HIV/AIDS, tuberculosis, and malaria (*22**,**23*). On the other hand, the public health component justifies including zoonoses such as bovine tuberculoses in current global programs and initiatives on tuberculosis control (*22*,*23*). Recognition of these facts should result in affected countries applying for funds from the Global Fund to Fight AIDS, Tuberculosis and Malaria (*24*). Surveillance and control of cross-border zoonotic diseases such as highly pathogenic avian influenza cannot be restricted to wealthy countries. According to Vallat, “One country not able to carry out early detection and rapid response to animal disease outbreaks can represent a threat to all the others” (*25*). To approach these threats, new partnerships (e.g., between resource-limited and industrial countries, public and private sectors, and animal and public health) and permanent dialogue are needed. “It is evident that the interest of the rich countries is to support the others in order to protect themselves” (*25*). Zoonosis control in general should thus be seen from a global perspective and lead to a call for a global subsidiary approach for control. International bodies like the World Organization for Animal Health, the Food and Agriculture Organization, and WHO should foster establishment of global standards for zoonosis surveillance and control. Fostering of global standards is also part of the WHO International Health Regulations that will come into force in mid-2007 and will require all countries to do a better job of surveillance for diseases that can spread between countries (www.int/gb/edwha/pdf_files/WHA58-REC1/english/Resolutions.pdf). These efforts should lead to a global fund for the control of zoonoses or become a component of an extended Global Fund to Fight AIDS, Tuberculosis and Malaria. Such a joint facility would allow coherent and integrated control approaches, particularly in the countries with the most serious resources constraints, which in turn would benefit the whole world.

## Conclusion

Zoonoses are among the most important animal and public health problems that affect the well-being of societies worldwide, yet they are too often forgotten or neglected. Because most zoonoses go unrecorded, they call for a rethinking of research and control efforts and the economic consequences. The example of brucellosis demonstrates that interventions in livestock against zoonoses, which would never be cost-effective when uniquely assessed from a public health sector point of view, may become cost-saving when considered from a societal perspective. Creating a new global finance facility for the control of zoonoses, similar to or linked with the Global Fund to Fight AIDS, Tuberculosis and Malaria, is timely, is of global interest, and represents a further contribution to successful attainment of the Millennium Development Goals.

## Appendix. Estimating Disability-adjusted Life Years

Disability-adjusted life years (DALYs) are used in the global comparative assessments of the burden of disease (*26*) and enable costs of interventions to be related to a standardized health outcome across diseases internationally. DALY is an indicator of the time lived with a disability and the time lost because of premature death (Formula 1).

DALY = years of life lost (YLL) + years of life with a disability (YLD) (1)

The duration of time lost due to premature death is calculated by using standard expected YLL with model life tables. The reduction in physical capacity due to illness is measured by using disability weights. To calculate the reduction in physical capacity, the following formula is used (Formula 2) (*26*):

where *a* is the age at onset of disease, *L* is the duration of disability or time lost due to premature mortality, *D* is the disability weight (or 1 for premature mortality), *r* is the discount rate, *C* is the age-weighting correction constant, and β is the parameter from the age-weighting function.

An estimate of the burden of disease for brucellosis is not readily available, so we therefore estimated the DALYs as a result of the disease by assuming that brucellosis is associated with a class II (0.2) disability weight (*D*), as the disease is perceived as very painful and affects occupational ability even during periods of remission (*27*). Average age at onset was calculated for every age group. For the duration of illness, we considered data by Beklemishev on the duration of clinical cure of 1,000 patients with brucellosis in the Russian Federation (*28*). The frequency distribution of clinical disease duration fits best with an exponential function for an average duration of 4.5 years. For duration of disease, we used @Risk (Palisade Corportation, Newfield, NY, USA) exponential function; β = 4.5 years. For cost-effectiveness, we used the median of the cumulated discounted DALYs, which corresponds to a median duration (*L*) of brucellosis of 3.11 years (*4*).
